# *In Vivo* Consequences of Disrupting SH3-Mediated Interactions of the Inducible T-Cell Kinase

**DOI:** 10.1155/2012/694386

**Published:** 2012-05-09

**Authors:** Roman M. Levytskyy, Nupura Hirve, David M. Guimond, Lie Min, Amy H. Andreotti, Constantine D. Tsoukas

**Affiliations:** ^1^Department of Biology, Molecular Biology Institute and Center for Microbial Sciences, San Diego State University, San Diego, CA 92182, USA; ^2^Department of Biochemistry, Biophysics, and Molecular Biology, Iowa State University, Ames, IA 50011, USA

## Abstract

ITK-SH3-mediated interactions, both with exogenous ligands and via intermolecular self-association with ITK-SH2, have been shown to be important for regulation of ITK activity. The biological significance of these competing SH3 interactions is not completely understood. A mutant of ITK where substitution of the SH3 domain with that of the related kinase BTK (ITK-BTK_(SH3)_) was used to disrupt intermolecular self-association of ITK while maintaining canonical binding to exogenous ligands such as SLP-76. ITK-BTK_(SH3)_ displays reduced association with SLP-76 leading to inefficient transphosphorylation, reduced phosphorylation of PLC*γ*1, and diminished Th_2_ cytokine production. In contrast, ITK-BTK_(SH3)_ displays no defect in its localization to the T-cell-APC contact site. Another mutation, Y511F, in the activation loop of ITK, impairs ITK activation. T cells expressing ITK-Y511F display defective phosphorylation of ITK and its downstream target PLC*γ*1, as well as significant inhibition of Th_2_ cytokines. In contrast, the inducible localization of ITK-Y511F to the T cell-APC contact site and its association with SLP-76 are not affected. The presented data lend further support to the hypothesis that precise interactions between ITK and its signaling partners are required to support ITK signaling downstream of the TCR.

## 1. Introduction

Protein tyrosine kinases play a critical role in signaling through the T-cell Antigen Receptor/CD3 (TCR-CD3) molecular complex [1]. The Tec family of tyrosine kinases regulates lymphocyte development, differentiation, and activation [2]. Among them, the Inducible T-Cell Kinase (ITK) plays an important role in T-cell signaling and function [3]. ITK is the major Tec kinase expressed in T cells, regulating intracellular Ca^++^ mobilization by phosphorylation and activation of Phospholipase C*γ*1 [4], TCR-induced actin polymerization [5], and production of Th_2_ cytokines [6]. Even though ITK does not appear to be important for the development of  Th_2_ cells per se, it is critical for the expression of Th_2_ cell effector function, as evidenced by defective expression of Th_2_ cytokines in ITK-deficient animals [7].

 ITK is structurally organized into domains that carry out its catalytic function, domains that regulate its catalytic activity, and domains that provide sites for interactions with other signaling partners [8]. Even though the crystal structure of full-length ITK has not been resolved, important insights into its structure have been provided by NMR-based analysis of individual ITK domains [2]. Previous *in vitro* observations provide some hints toward a more complete understanding of the mechanistic role of specific domains within ITK [8]. There remains a need, however, for further advances in our understanding of the role of specific ITK domains in live cells.

 The ITK SH3 domain has been shown to mediate more than one protein-protein interaction through its conserved binding site. Namely, ITK SH3 binds to the proline-rich region of SLP-76 as well as to the SH2 domain of another ITK molecule [9–12]. The latter interaction does not involve a canonical proline-rich site on the ITK SH2 domain but nevertheless is mediated by the conserved SH3 binding groove [12]. Thus, the ITK SH3 binding groove participates in mutually exclusive interactions. An ITK variant where the ITK-SH3 domain has been replaced with the SH3 domain of the related kinase BTK (henceforth designated ITK-BTK_(SH3)_) was designed to block the intermolecular interaction between ITK SH3 and SH2 domains while retaining binding to a proline-rich ligand [13]. Previously, a group of us (LM, AHA) had independently studied the properties and biological effects of this ITK variant using primarily a heterologous nonlymphoid cellular system [13]. In the present investigation, we have revisited this issue by using a more relevant lymphoid system. We find that transfection of this mutant construct into Jurkat T cells and ITK-deficient mouse lymphoid cells displays significant effects on the transphosphorylation of ITK on tyrosine 511, the subsequent phosphorylation of the downstream target PLC*γ*1, and on the production of TCR-induced Th2 cytokines. The loss of phosphorylation on Y511 and subsequent downstream events mimic the signaling defects observed using direct mutation of Y511 (ITK-Y511F). In contrast, neither variant affects the inducible localization of ITK to the T cell-APC contact site, and differential effects on the ability of ITK to associate with its intracellular signaling partner SLP-76 are observed.

## 2. Materials and Methods

### 2.1. Cells and Reagents

The Jurkat subline, JTag, was a kind gift from Dr. Amnon Altman (La Jolla Institute of Allergy and Immunology, La Jolla, CA). Raji cells (CCL-86) were obtained from the American Type Culture Collection. Cells were cultured in RPMI 1640 (Mediatech) supplemented with 10% FBS, 2 mM L-Glutamine, 100 U/mL penicillin, 100 *μ*g/mL streptomycin, 20 mM HEPES (all obtained from Mediatech) in a 37°C humidified incubator in 5% CO_2_ atmosphere. Thymocytes were isolated from ITK-deficient (Itk^−/−^) mice that were obtained from Dr. D. Littman (New York University School of Medicine) and bred in our own animal facility. Mice were male or female and used at 6–12 weeks of age. All experimental protocols using animals were approved by the IACUC of San Diego State University.

### 2.2. Antibodies

Anti-human CD3*ε* monoclonal antibody OKT3 was prepared in house from a hybridoma (CRL8001) obtained from ATCC. Anti-mouse CD3*ε* (2C11; cat number 553058) and anti-mouse CD28 (37.51; cat number 553295) were obtained from BD Pharmingen. Rabbit polyclonal anti-ITK antibody (cat number 06-546; Upstate) was used for immunoprecipitation and mouse monoclonal anti-ITK antibody (clone 2F12; Upstate) was used for immunoblotting. Antiphosphotyrosine (clone 4G10) antibody was from Upstate and rabbit anti-SLP-76 IgG (cat number 4958) was from Cell Signaling Technology. Anti-GFP (mixture of clones 7.1 and 13.1) antibody was obtained from Roche. For phosphoflow cytometry, we used Alexa 647-conjugated antibodies to ITK pY511 (clone 24a/BTK), PLC*γ*1 pY783 (clone 27/PLC), and ZAP-70 pY319 all obtained from BD Biosciences. Additional antibodies used in these studies were DyLight 405-conjugated anti-Golden Syrian and Armenian Hamster antibody (cat number 620-146-440; Rockland Immunochemicals) and Goat anti-Armenian Hamster (cat number 127-005-160; Jackson ImmunoResearch).

### 2.3. DNA Constructs

**Figure 1:**
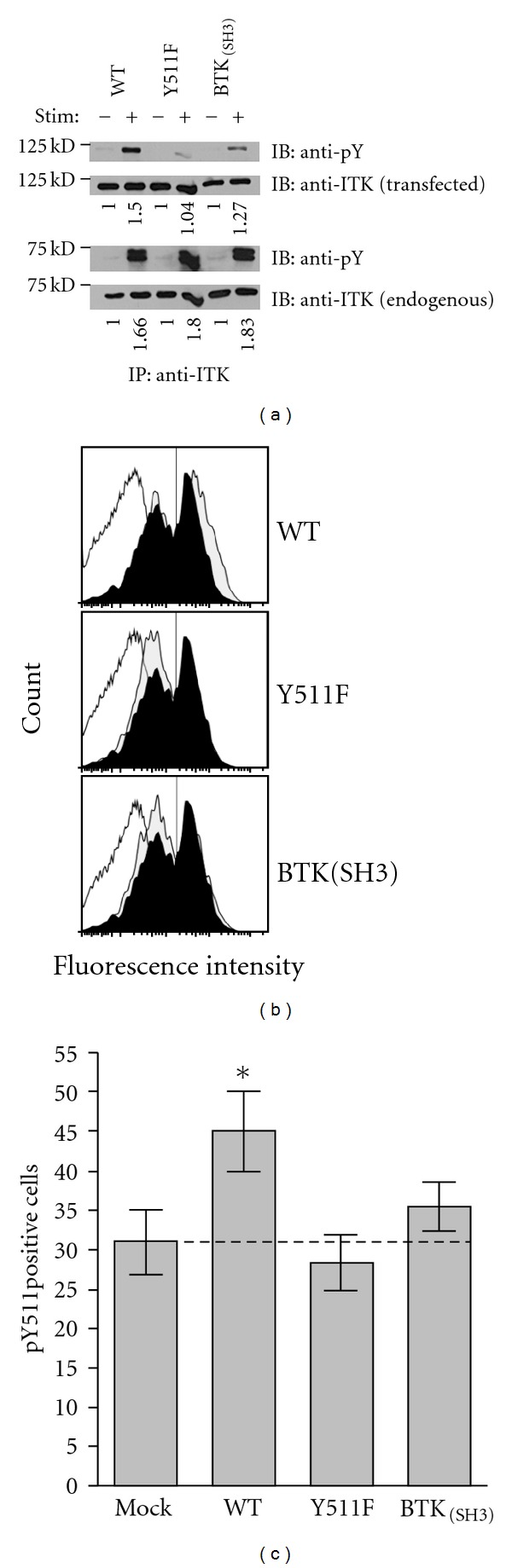
Effects of ITK-Y511F and ITK-BTK_(SH3) _ mutations on ITK phosphorylation. (a) Jurkat cells that had been transfected with the indicated ITK constructs were stimulated (+) or not (−) with anti-CD3*ε* antibodies and then lysed. ITK in the lysates was immuno-precipitated (IP) with anti-ITK antibodies, immune complexes resolved by SDS-PAGE, proteins transferred onto PVDF membranes and immuno-blotted (IB) sequentially with antiphosphotyrosine antibodies and anti-ITK antibodies as indicated. Bands were visualized by chemiluminescence as described in Section 2. Numbers under the panels represent the fold increase in band signal intensity (phosphorylation) compared to the respective nonstimulated control calculated as described in Section 2. Results are those from one of three replicate experiments with similar results. (b) Murine thymocytes from ITK-deficient mice, nucleofected with the indicated ITK constructs, were stimulated with anti-CD3*ε* antibodies for 1 minute and then analyzed for ITK phosphorylation by flow cytometry using Alexa 647-tagged antibodies that react with ITK pY511, as described in Section 2. Results are displayed as cell number (linear scale) versus fluorescence intensity (logarithmic scale). In each panel the open histograms represent nucleofected/nonstimulated cells, the black filled histograms nonnucleofected/stimulated cells, and the grey filled histograms nucleofected/stimulated cells. The cDNA's used in each nucleofection is indicated to the right of each panel. The vertical line denotes the electronic gate used to calculate percent positive cells. The results are those of one representative experiment. (c) Average (±SEM) percentage of pY511 positive cells of three replicate experiments (including the experiment depicted in panel (b) performed as described in panel (b). The * denotes that the average of pY511 positive cells in WT-ITK nucleofected group is significantly different from the rest of the groups at *P* < 0.05 (student's *t* test). The dotted line demarcates the specific anti-pY511 reactivity.

**Figure 2:**
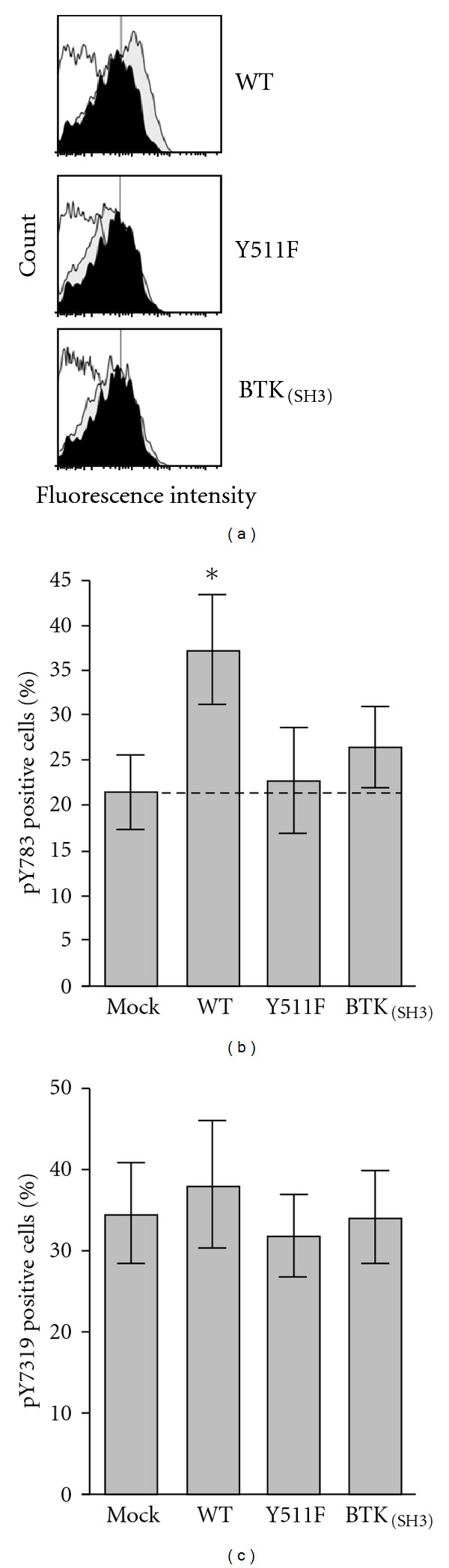
Reduced transphosphorylation of PLC*γ*1 by ITK-Y511F and ITK-BTK_(SH3) _ mutants. (a) murine thymocytes from ITK-deficient mice, nucleofected with the indicated ITK constructs, were stimulated with anti-mouse-CD3*ε* antibodies and then analyzed for PLC*γ*1 phosphorylation using Alexa 647-tagged antibodies that react with PLC*γ*1 pY783, as described in Section 2. Results are displayed as cell number (linear scale) versus fluorescence intensity (logarithmic scale). In each panel, the open histograms represent nucleofected/nonstimulated cells, the black filled histograms nonnucleofected/stimulated cells, and the grey filled histograms nucleofected/stimulated cells. The cDNA's used in each nucleofection is indicated to the right of each panel. The vertical line denotes the electronic gate used to calculate percent positive cells. The results are those of one representative experiment using the same cells as in Figure 1(b). (b) Average (±SEM) percentage of pY783 positive cells of three replicate experiments (including experiment in panel (a) performed as described in panel (a). The cells used in these experiments are the same as those used in Figure 1(c). The * denotes that the average of pY783 positive cells in WT-ITK nucleofected group is significantly different from the rest of the groups at *P* < 0.05 (Student's *t* test). The dotted line demarcates the specific anti-pY783 reactivity. (c) Average (±SEM) percentage of pY319 positive cells of four replicate experiments performed as described in panel (a), but analyzed for ZAP-70 phosphorylation using Alexa 647-tagged antibodies that react with ZAP-70 pY319.

**Figure 3:**
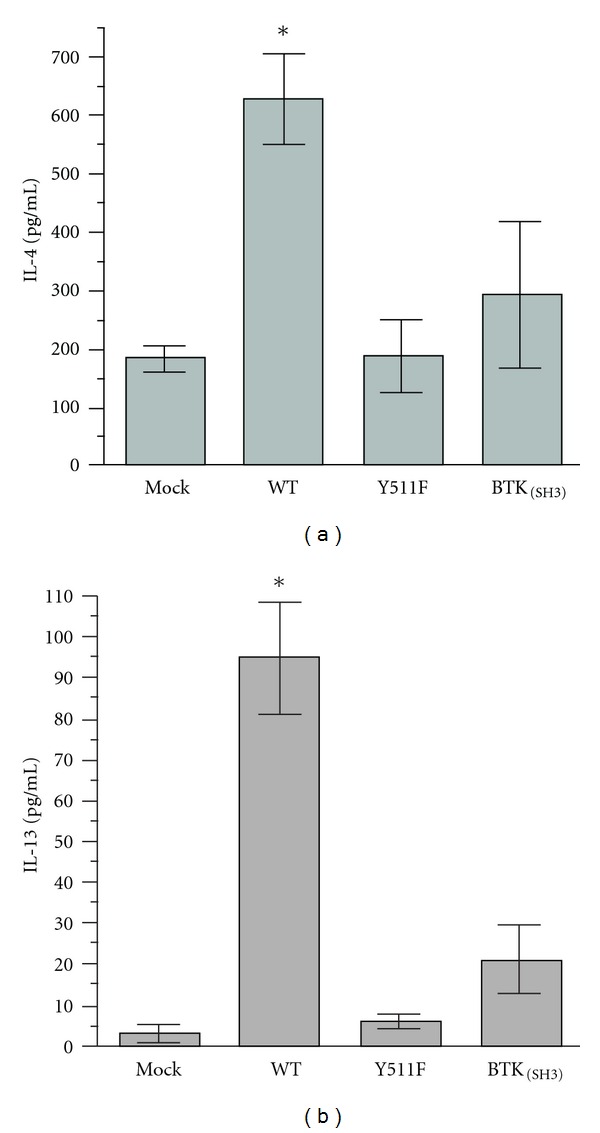
Deficient Th2 cytokine production by ITK-Y511F and ITK-BTK_(SH3) _ mutants. Murine thymocytes were nucleofected with the indicated ITK constructs and then cultured under Th2 skewing conditions as described in Section 2. Culture supernatants were assayed in duplicate for IL-4 (a) and IL-13 (b) using commercial ELISA assay kits and following manufacturer's recommendations. Results are displayed as the averages (±SEM) of three replicate experiments for IL-4 and two replicate experiments for IL-13. Negative controls (nonstimulated cell-culture supernatants) contained 69 pg/mL IL-4 and undetectable (<4 pg/mL) IL-13.

**Figure 4:**
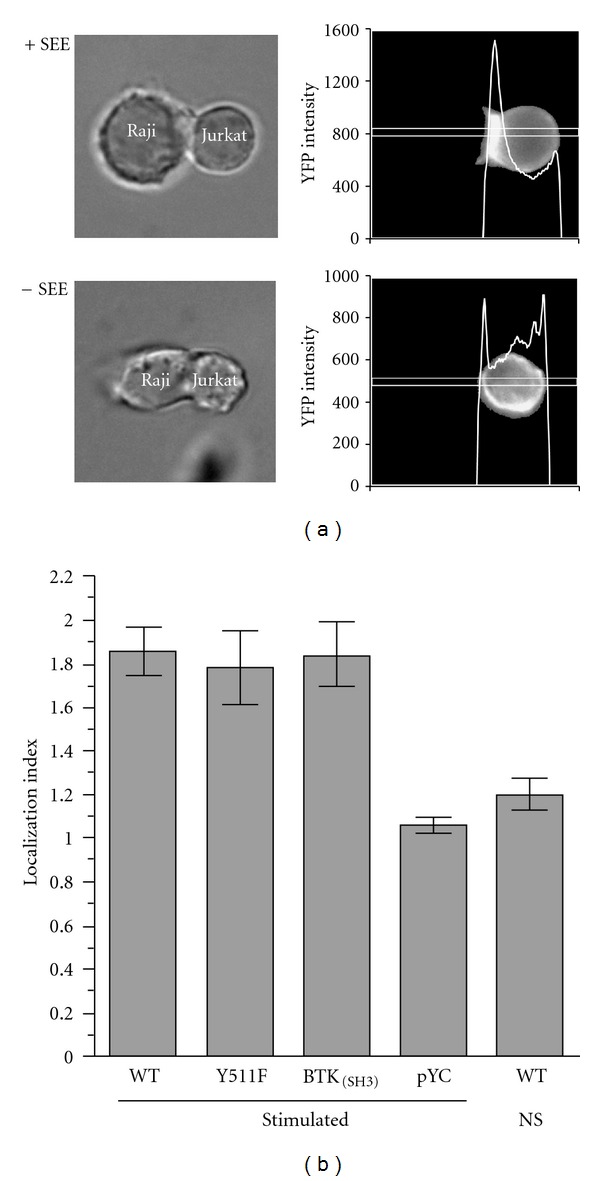
Inducible localization of ITK-Y511F and ITK-BTK_(SH3) _ to the T-cell-APC contact site. Jurkat cells transfected with fluorescent protein chimeric constructs of WT-ITK, ITK-Y511F, or ITK-BTK_(SH3) _ or with a construct containing fluorescent protein without ITK (pYC), as a transfection control, were incubated with Raji cells that had been pretreated (+SEE) or not treated (−SEE) with SEE for 3 minutes at 37°C. Cells were then fixed in paraformaldehyde and analyzed using epifluorescence microscopy. (a) Representative experiment displaying localization of ITK as YFP fluorescence intensity (right hand panels) in the presence (top panels) or absence (bottom panels) of SEE. Differential interference contrast images are displayed in the left hand panels for ease of orientation. (b) Results of three replicate experiments, performed, and analyzed as in (a), are displayed as the average Localization Index (±SEM) for each ITK construct calculated as described in Section 2. WT/NS denotes nonstimulated cells transfected with WT-ITK, as negative control. The number of conjugates analyzed in each group are WT, *N* = 52; Y511F, *N* = 37; BTK_(SH3)_, *N* = 32; pYC, *N* = 10, WT/NS; *N* = 10.

**Figure 5:**
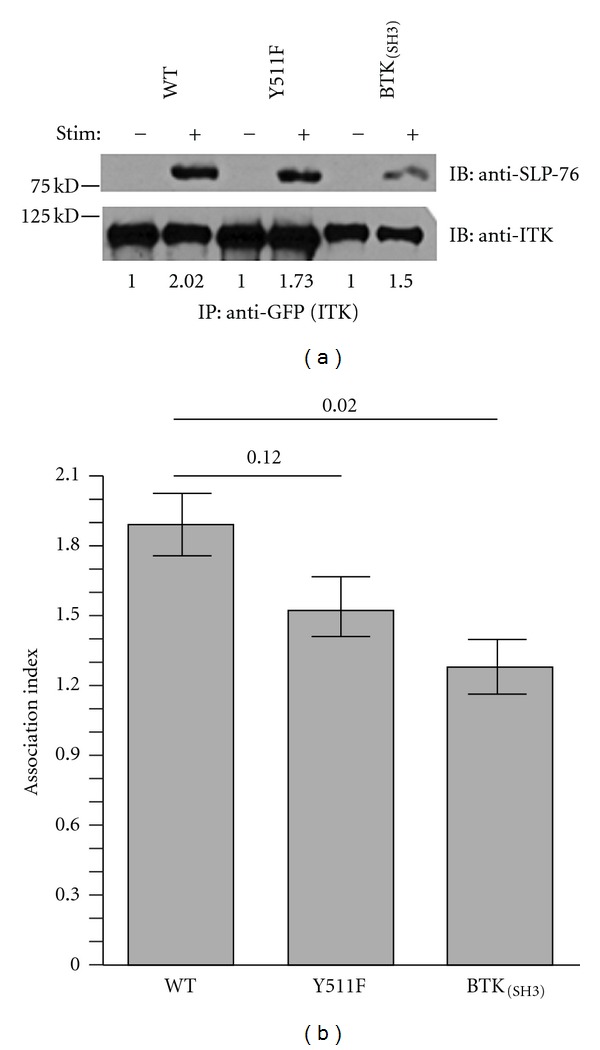
Association of ITK-Y511F and ITK-BTK_(SH3) _ with SLP-76. (a) Jurkat cells that had been transfected with the indicated GFP-ITK constructs were stimulated (+) or not (−) with anti-CD3*ε* antibodies and then lysed. ITK in the lysates was immunoprecipitated (IP) with anti-GFP antibodies, immune complexes resolved by SDS-PAGE, proteins transferred onto PVDF membranes, and immunoblotted (IB) sequentially with anti SLP-76 (top panel) and anti-ITK (bottom panel) antibodies. Bands were visualized by chemiluminescence as described in Section 2. Numbers under each blot represent the association index between each ITK-variant and SLP-76 calculated as described in Section 2. Results are those from one of three replicate experiments. (b) average (±SEM) ITK-SLP-76 association index of three replicate experiments (including the experiment depicted in panel (a) performed as described in panel (a). Numbers in brackets denote *P* values calculated by the Student's *t* test.

For the data displayed in Figures 1(a), 4, and 5, where the JTag T-cell line was utilized, Fluorescent Protein (FP) chimeric cDNA constructs of ITK-WT, ITK-Y511F, and ITK-BTK_(SH3)_ were expressed into the pME18s vector and transfected as previously described [14]. For the data displayed in Figures 1(b), 1(c), 2, and 3, where primary mouse thymocytes were utilized, GFP-chimeric ITK genes were expressed in a pCMV-driven vector (Clontech). The ITK-BTK_(SH3) _ variant has been previously described [13]. The ITK-Y511F point mutant, created by using the GeneTailor site-directed mutagenesis kit (Invitrogen), was originally described by Heyeck et al. [15]. pYC, a FP-cDNA that contains no ITK, was used as negative control. It was kindly provided to us by Dr. Tomaz Zal (MD Anderson) and it has been previously described [16].

### 2.4. Treatment with Staphylococcal Enterotoxin E (SEE) and Conjugate Formation

Raji cells, labeled with Cy5-ester (Amersham), were preincubated with 10 *μ*g/mL SEE (Toxin Technology) for 2 hours at 37°C. Following washing, they were mixed with equal numbers (1 × 10^6^) of JTag cells that had been transfected with various ITK-expression vectors. Cells were mixed, pelleted, and incubated in a total volume of 200 *μ*L for 3 minutes at 37°C. They were then immediately fixed with an equal volume of 4% paraformaldehyde (10 minutes on ice), washed, resuspended in 40 *μ*L PBS, mounted on slides, and analyzed by epifluorescence microscopy (AxioVision Imaging System, Carl Zeiss) with 0.5 Neutral Density filter. Morphological conjugates were selected by DIC using stable conjugate criteria as previously described [17]. The Localization Index was calculated as previously described [11] using the NIH ImageJ software. Briefly, a transect across the T cell was drawn, as shown in Figure 4(a), and the ratio of pixel intensity (FP-ITK) at the contact site was divided by that of the opposite noncontact site.

### 2.5. Transfection, Cell Stimulation, Immunoprecipitation, and Immunoblotting

These procedures were performed as previously described [11]. For coimmunoprecipitation experiments, Jukat cells, 20 × 10^6^ per mL, were stimulated with 5 *μ*g/mL of anti-CD3*ε* antibody OKT3 that was cross-linked with an equal amount of rabbit anti-mouse IgG and incubated for 1 minute at 37°C. Cells were then lysed and FP-ITK was immunoprecipitated with anti-GFP IgG (per supplier's recommendation), immune complexes were resolved in 7.5% SDS-PAGE, and SLP-76 and ITK were immunoblotted on PVDF membranes (Pall Life Sciences) with the respective specific antibodies at 1 *μ*g/mL. Signals were detected by HRP-conjugated goat anti-mouse IgG (Jackson Immunoresearch) using the SuperSignal West Pico Chemiluminescence Substrate (Pierce) per manufacturer's directions. The ITK-SLP-76 Association Index was calculated by densitometric analysis using the NIH ImageJ software. Briefly, pixel intensity of each lane was divided by the pixel intensity of its respective loading control lane, then each obtained ratio was divided by the ratio of the respective nonstimulated lane. Phosphorylation analysis was performed similar to above. ITK was immunoprecipitated by anti-ITK IgG (10 *μ*g/mL) and phospho-ITK was immunoblotted by antiphosphotyrosine (4G10) IgG (2 *μ*g/mL) and detected by chemiluminescence as above. The fold change in phosphorylation of each ITK mutant upon stimulation conditions was calculated as described above for the coimmunoprecipitation experiments.

### 2.6. Nucleofection

Thymocytes were isolated from the thymi of ITK-deficient mice by separation through a nylon mesh. Cells, 10 × 10^6^, were nucleofected with 15 *μ*g DNA of the various ITK-constructs in 100 *μ*L volume using the reagents provided by the manufacturer (Lonza) and the Amaxa Nucleofector II (program X-001) following the manufacturer's instructions. The nucleofected cells were then cultured in 1.5 mL of Lonza Nucleofector medium supplemented with 10% FBS at 37°C in a humidified 5% CO_2_ atmosphere for 24 hours following the manufacturer's instructions, before used in experiments. An aliquot of the cells were mock nucleofected by treating them identically to the above, but in the absence of DNA.

### 2.7. Cell Stimulation and Phosphoflow Cytometry

Thymocytes were stimulated and analyzed for intracytoplasmic phosphorylation using a modification of a previously published method [18, 19]. Following nucleofection with GFP-ITK cDNA constructs thymocytes were resuspended in 500 *μ*L of ice-cold medium containing Armenian Hamster anti-mouse CD3*ε* (1 *μ*g/mL) antibody and incubated for 1 hour on ice. Cells were then pelleted and resuspended in 500 *μ*L of ice-cold medium containing a premixed cocktail of 7 *μ*g goat anti-Armenian Hamster antibody and 0.5 *μ*g of DyLight 405-conjugated anti-Golden Syrian and Armenian Hamster antibodies and incubated for an additional 30 minutes on ice. The samples were then stimulated by placing them in a 37°C water bath for 1 minute and immediately fixed by adding an equal volume of ice-cold paraformaldehyde (4%). Following fixation, cells were resuspended in 1 mL of ice cold 95% methanol and incubated overnight at 4°C. After several washes with PBS-0.5% BSA, samples were blocked by incubation with 100 *μ*g/mL of mouse IgG (1 hour RT°) and stained with the respective Alexa 647-conjugated anti-phospho-specific antibodies (1 hour, room temperature, in the dark) following the manufacturer's recommendation. Following several washes, the cells were analyzed using a FACSAria flow cytometer (BD Biosciences) and FlowJo 7 (FlowJo) software. In parallel cultures, control nonstimulated thymocytes were treated as above, but in the absence of anti-CD3*ε*. In order to analyze phosphoflow signals in stimulated cells, we first established an electronic gate encompassing GFP-positive (transfected) cells using mock-transfected cells as controls. Cells included in that gate were further gated for the top 15% MFI of DyLight 405 signal (anti-CD3*ε* positive; defined as stimulated cells). Cells within this gate were analyzed for specific relevant Alexa 647 signal (phosphotyrosine-positive cells) and plotted as cell number versus Alexa 647 fluorescence intensity.

### 2.8. Skewing, Cytokine Production, and Measurement

In order to increase the frequency towards Th2 cytokine production, thymocytes nucleofected as described above were skewed using modifications of previously published protocols [7, 20, 21]. Briefly, thymocytes were collected 4 hrs after nucleofection and resuspended at 2 × 10^6^ cells per mL in Lonza Medium-10% FBS containing anti-CD28 (1 *μ*g/mL) and anti-IL12 (20 *μ*g/mL) antibodies. Cells were placed into 96-well U-bottom well plates (2 × 10^5^ per well) that had been precoated with anti-CD3*ε* antibody 2C11 (1 *μ*g/mL) and incubated overnight at 37°C in a humidified incubator with 5% CO_2_ atmosphere. Cells were then supplemented with IL2 (5 ng/mL) and IL4 (10 ng/mL) and incubated for an additional 2 days. Cells were then resuspended in fresh medium containing anti-CD28 (1 *μ*g/mL) antibody and transferred to new anti-CD3*ε* coated plate wells and incubated for 24 hours as above. Cell-free culture supernatants were assayed for IL-4 and IL-13 using commercially obtained ELISA kits. The limit of detection in the IL-4 kit (BD Bioscience, cat number 555232) is 8 pg/mL and for the IL-13 kit (eBioscience, cat number 88-7137-88) is 4 pg/mL. Analysis was performed in duplicate wells of 96-well ELISA plates (BD Bioscience) following the manufacturer's recommendations and read on eMax Precision Microplate Reader using SoftMax Pro v5.0 software (Molecular Devices).

## 3. Results

### 3.1. Effect of ITK-BTK_**(SH3)**_ Mutation on ITK Phosphorylation

Activation of ITK depends on transphosphorylation of tyrosine 511 in the activation loop of the kinase [15, 22]. Using Jurkat T cells transfected with either wild-type ITK, a Y511F-ITK mutant, or ITK-BTK_(SH3)_, we first assessed ITK phosphorylation levels following stimulation through the TCR (Figure 1(a)). As expected, mutation of tyrosine 511 to phenylalanine precluded TCR-mediated phosphorylation of ITK in Jurkat T cells; TCR-induced phosphorylation signal of ITK-Y511F transfectants was similar to that of nonstimulated controls (Figure 1(a), top panel). In contrast, ITK-BTK_(SH3) _ was phosphorylated, albeit at lower levels than wild type ITK (Figure 1(a), top panel). In the displayed experiment there was a reduction of about 15% in signal intensity.

 Since Jurkat cells express endogenous ITK that may affect the outcome, we wished to confirm the above results more quantitatively in a cellular system devoid of ITK. To this end, we nucleofected thymocytes obtained from ITK-deficient mice with the three ITK constructs (wild type, Y511Y, and ITK-BTK_(SH3)_) and quantified TCR-mediated phosphorylation of ITK by phospho-flow cytometry using an antibody that reacts with ITK-phosphotyrosine 511. Since an antibody made specifically against ITK-pY511 is not available, we used an antibody that was generated against the analogous amino acid (pY551) of the related kinase BTK and cross-reacts with ITK pY511 [23]. We observed that mock- or nonnucleofected, TCR-stimulated thymocytes from ITK-deficient mice displayed significant reactivity (30%) upon staining with this antibody (Figure 1(b), dotted line open histogram and Figure 1(c)). Thus, it appears that this antibody has reactivity to other phosphotyrosine residues, most likely those of other Tec kinases present in the cells (i.e., TEC and RLK). Importantly, however, upon nucleofection with WT-ITK there was a fifty percent increase in the phosphosignal (Figure 1(b), grey histogram in top panel and Figure 1(c)) that was significant at *P* < 0.05 (Figure 1(c), comparison between Mock and WT groups). In contrast, no significant increase from Mock- or nonnucleofected cells occurred in thymocytes nucleofected with either ITK-Y511F or ITK- BTK_(SH3) _ (Figures 1(b) and 1(c)). It should be noted that the analyzed cells in all groups displayed equivalent expression of the nucleofected genes as assessed by the fluorescence intensities (MFI signal) of the GFP-nucleofected constructs. In the representative experiment shown in Figure 1(b) the MFI values for the nucleofected cDNA's were WT:1940, Y511F:1920, and BTK_(SH3)_:1984. Similarly equivalent were the MFI's for the three replicate experiments shown in Figure 1(c). Thus, while ITK-BTK_(SH3) _ appears to be phosphorylated in transfected Jurkat cells, albeit at lower levels compared to WT-ITK, this variant fails to be phosphorylated on Y511 when nucleofected into thymocytes derived from ITK-deficient mice. One interpretation of this observation could be that the phosphorylation of ITK-BTK_(SH3) _ we see in the Jurkat cell system (Figure 1(a)) might be due to the possible interaction between the endogenous ITK-SH3 domain with the SH2 domain of the transfected ITK-BTK_(SH3)_.

### 3.2. Reduced Transphosphorylation of PLC*γ*1 by ITK-Y511F and ITK-BTK_**(SH3)**_ Mutants

Previous studies have shown that upon TCR engagement, ITK phosphorylates PLC*γ*1 on tyrosine 783 [4]. This is a critical phosphorylation target for the activation of PLC*γ*1 and downstream signaling events [4]. To determine the ability of ITK-Y511F and ITK-BTK_(SH3) _ to phosphorylate PLC*γ*1 on Tyr 783, we nucleofected thymocytes from ITK-deficient mice with the two mutant constructs and compared the inducible phosphorylation of PLC*γ*1-Y783 by phosphoflow cytometry using a specific anti-pY783 antibody. It is interesting that TCR-stimulated mock- or nonnucleofected thymocytes displayed significant pY783 phosphorylation (Figures 2(a) and 2(b)). Since the cells lack ITK and the specificity of the anti-pY783 reactivity has been previously documented [24], we interpret this finding as indicating that other kinases are able to phosphorylate PLC*γ*1 at this critical residue. Importantly, however, upon nucleofection with WT-ITK, the phosphosignal nearly doubles indicating increased phosphorylation of PLC*γ*1 Y783 specifically by wild-type ITK (Figures 2(a) and 2(b)). Compared to mock- or nonnucleofected cells, this increase is significant at *P* < 0.05. In contrast, nucleofection with either ITK-Y511F or ITK-BTK_(SH3) _ did not significantly increase phosphorylation above the mock-transfected controls. The specificity of the PLC*γ*1 Y783 phosphorylation event is further demonstrated by the fact that none of these transfectants had any effect on the phosphorylation of the upstream signaling molecule ZAP-70 (Figure 2(c)). The cells used for the of PLC*γ*1 phosphorylation analysis were aliquots from the same cells used in the experiments of Figures 1(b) and 1(c). As such, they displayed equivalent expression of the nucleofected genes as described above.

### 3.3. Deficient Th_2_ Cytokine Production by ITK-Y511F and ITK-BTK_**(SH3)**_ Mutants

During the course of analyzing ITK and PLC*γ*1 phosphorylation in thymocytes from ITK-deficient mice, we also examined Th_2_ cytokine production in the context of wild-type and variant ITKs. ITK is critical for the transcriptional activation of the physically clustered Th_2_ cytokine genes IL-4 and IL-13 [25]. Therefore, we wanted to determine whether the transphosphorylation-deficient (ITK-Y511F) and the ITK-BTK_(SH3) _ variant could affect the production of these two cytokines upon TCR-stimulation. To accomplish this, we skewed the nucleofected thymocytes towards Th_2_ cytokine production in order to increase the relative frequency of the cells producing these cytokines. Compared to WT-ITK nucleofected thymocytes, those nucleofected with either of the two mutant genes (ITK Y511F or ITK-BTK_(SH3)_) produced background cytokine levels similar to those seen with mock-transfected controls (Figures 3(a) and 3(b)).

### 3.4. Inducible Localization of ITK-BTK_**(SH3)**_ and ITK-Y511F to the T-Cell-APC Contact Site

All of the results described above (Figures 2 and 3) for the ITK Y511F mutant and the ITK-BTK_(SH3) _ variant are consistent with the observed lack of phosphorylation on Y511 within ITK (Figure 1). It was expected that the ITK Y511F mutant would lack phosphorylation on Y511 but the finding that the ITK-BTK_(SH3) _ variant is also poorly phosphorylated on Y511 when nucleofected into thymocytes derived from ITK-deficient mice was not anticipated. To better understand why the ITK-BTK_(SH3) _ variant is not efficiently phosphorylated on Y511, we next examined localization of wild-type and mutant ITKs to the T cell-APC contact site and more specifically association of these ITK mutants with SLP-76. It has been previously demonstrated that upon T cell activation, ITK colocalizes with the TCR-CD3 complex at the APC-T cell contact site [14, 26]. Whether ITK requires transphosphorylation on its Tyr 511 before its translocation to the contact site has not been previously addressed. Moreover, we wished to ascertain whether the poor phosphorylation on Y511 in the ITK-BTK_(SH3) _ variant might be due to improper localization. The data in Figure 4 clearly demonstrate that both ITK Y511F and ITK-BTK_(SH3) _ localize in a manner that is similar to wild-type ITK in Jurkat cells. For the ITK Y511F mutant in particular, it can be concluded that phosphorylation at this site is not a prerequisite for inducible localization to the T cell-APC contact site. The data also indicate that the ITK-BTK_(SH3) _ variant performs as well as wild-type ITK; the diminished self-association of the ITK-BTK_(SH3) _ variant, as well as the observed decrease in phosphorylation of Y511 (Figure 1), do not affect its ability to inducibly localize to the APC-T-cell contact site (Figure 4). Upon stimulation, WT-ITK displayed approximately two-fold increase in its localization index that was similar to that seen in both Y511F and ITK-BTK_(SH3)_ transfectants (Figure 4(b)). Control, nonstimulated cells transfected with WT-ITK or with a construct containing fluorescent protein without ITK (pYC) displayed insignificant localization (Figure 4(b)).

### 3.5. Association of ITK-Y511F and ITK-BTK_**(SH3)**_ with SLP-76

We next specifically examined the SH3-mediated interaction of ITK following TCR stimulation since the ITK-BTK_(SH3) _ variant carries a nonnative SH3 domain. It has been previously demonstrated that TCR-induced activation results in ITK association with the adaptor protein SLP-76 [10, 11]. To address whether ITK-BTK_(SH3) _ maintains normal SLP-76 association, ITK (wild type, ITK-BTK_(SH3)_, or ITK Y511F) was immunoprecipitated from lysates of cells that had been transfected with these mutants and stimulated through the TCR. SLP-76 coimmunoprecipitation was assessed by blotting with an anti-SLP-76 antibody (Figure 5). The results demonstrate that the Y511F mutation does not significantly affect the inducible association of ITK with SLP-76 (Figures 5(a) and 5(b)). However, the association of the ITK-BTK_(SH3) _ variant with SLP-76 is significantly reduced (~32% Figures 5(a) and 5(b).

## 4. Discussion

Previous NMR-based analysis uncovered a novel intermolecular association between the SH2 and SH3 domains of ITK that does not involve conventional proline-rich motifs [12]. Instead, this association involves the interaction of the ITK SH3 binding pocket with four loops of the ITK SH2 domain located at a site distinct from the phosphotyrosine binding site of this domain [12]. The SH2/SH3 ITK domain interaction appears to be specific, as these domains do not interact with the respective domains of non-Tec kinases, thus suggesting a functionally important role [12]. Moreover, the ITK SH2 domain does not interact with BTK SH3, the domain most closely related to ITK SH3. These observations inspired the construction of a variant of ITK where the SH3 domain was replaced with that of BTK [13]. Even though this ITK variant (ITK-BTK_(SH3)_) retained the ability to bind a canonical proline-rich peptide ligand, it displayed limited ability to interact with the ITK SH2 domain in an intermolecular fashion, thus becoming a useful reagent for studying the functional role of intermolecular ITK self-association [13].

 In the past, a group of us (LM, AHA), through a series of *in vitro *assays, independently found that the ITK-BTK_(SH3) _ variant displays reduced self-association compared to the wild-type ITK molecule, maintains normal binding to a proline-rich peptide ligand derived from SLP-76, and displays normal phosphorylation of Tyr 511 in its activation loop that is critical for the enzymatic activation of ITK [15, 22]. Furthermore, at higher concentration, ITK-BTK_(SH3) _ was found to exhibit increased catalytic activity towards its target PLC*γ*1 compared to wild-type ITK at the same concentration, led to increased ERK phosphorylation, and increased calcium flux compared to wild-type ITK [13]. Since these previous studies were performed *in vitro* using a primarily heterologous nonlymphoid system and retroviral transduction, we determined that further analysis of the ITK-BTK_(SH3) _ variant was warranted. Therefore, in the present investigation, we have revisited this issue using a more relevant lymphoid cellular system.

 When human Jurkat T cells were transfected with ITK-BTK_(SH3) _ and the inducible phosphorylation of Tyr 511 was assessed, we found a different result than the one described above. Namely, a decrease in transphosphorylation of Tyr 511 (Figure 1(a)). To confirm this in a more physiological target, we nucleofected primary mouse lymphoid cells (thymocytes) with ITK-BTK_(SH3) _ and assessed inducible phosphorylation of Tyr 511 by phosphoflow cytometry. The data again indicate that Tyr 511 phosphorylation of ITK-BTK_(SH3) _ was significantly lower than WT-ITK and almost as low as that observed for the ITK Y511F mutant. The subsequent observations that downstream PLC*γ*1 phosphorylation and IL-4 and IL-13 production are significantly reduced are completely consistent with the defective phosphorylation of Tyr 511 since ITK is not activated and does not phosphorylate PLC*γ*1 unless the ITK activation loop tyrosine (Y511) is phosphorylated [15, 22].

 In an effort to understand why the ITK-BTK_(SH3) _ variant fails to get phosphorylated on Y511 in the nucleofected lymphoid system, we have focused on its assembly into the SLP-76/LAT signaling complex. Multiple interactions mediated by different domains of ITK serve to colocalize ITK with its signaling partners [9, 10, 27, 28]. Our data in Figures 4 and 5 suggest that while the ITK-BTK_(SH3) _ variant inducibly localizes to the T cell-APC contact site in a manner that is indistinguishable from wild-type ITK, the specific SH3-mediated interaction of ITK with SLP-76 is not maintained in the ITK-BTK_(SH3) _ variant.

 Previous results indicate that a short model peptide (residues 184–195 of SLP-76), representing the minimal binding site of the SLP-76 polyproline-rich region to ITK-SH3 domain [9], has nearly identical binding affinity to both ITK- and BTK-SH3 domains [13]. Based on these data, we expected that ITK-BTK_(SH3) _ would exhibit normal binding to SLP-76. However, the data presented here do not bear this out, ITK-BTK_(SH3) _ does not associated with SLP-76 to the same extent as wild-type ITK (Figure 5). Thus, the short SLP-76 proline-rich peptide, while sufficient for inhibition of the TCR-induced association between ITK and SLP-76 both *in vitro* and *in vivo* [11], may not be a good model for the association between full-length ITK-BTK_(SH3) _ and full-length SLP-76. It is possible that the interaction between the SH3 domain of ITK and SLP-76 extends beyond the canonical binding motif within SLP-76 and cannot be accurately mimicked using a simple linear proline-rich peptide ligand. Amino acid sequence differences between the ITK- and BTK-SH3 domains might then manifest in the reduced association of ITK-BTK_(SH3) _ with SLP-76. Thus, in view of the lack of complete homology between the ITK- and BTK-SH3 domains, it is possible that additional residues beyond the minimal amino acid sequence of the short peptide may more accurately reflect the complete ITK binding site that would show affinity differences between ITK and BTK SH3 domains in an *in vitro *binding assay. 

 In the context of the cellular system developed here, we suggest that diminished binding between ITK and SLP-76 is responsible for the reduced activation of ITK as measured by phosphorylation on Y511. This is consistent with previous work showing that specific disruption of the ITK SH3/SLP-76 interaction with a cell permeable peptide targeted to the ITK SH3 domain results in inhibition of transphosphorylation of ITK on Y511 [11]. Loss of Y511 phosphorylation in turn explains the reduced phosphorylation of PLC*γ*1 and reduced cytokine production. The question remains why the system developed by Min et al. showed increased PLC*γ*1 activation (as measured by calcium flux) and increased downstream signaling (as measured by ERK phosphorylation).

 A possible explanation for the disparate results between the earlier work of Min et al. and the findings reported here might lie in levels of protein expression in the different systems used to examine the behavior of the ITK-BTK_(SH3) _ variant. It is possible that the retroviral expression system used by Min et al. produced levels of ITK protein in the ITK^−/−^ T cells that were sufficient to overcome the reduced affinity of the ITK-BTK_(SH3) _ variant for SLP-76 that has now become apparent in the current study. Increased protein levels would drive the binding event toward SLP-76 associated ITK-BTK_(SH3) _ to the extent that Y511 phosphorylation levels would not be adversely affected and the enhanced activity of the ITK-BTK_(SH3) _ variant (presumably increased due to loss of self-association) leads to increased signaling when compared to transduction of the same levels of wild-type ITK in the same system. In the current work, we were unable to assess the effect of altered self-association of the ITK-BTK_(SH3) _ variant since the required activation of ITK by transphosphorylation of Y511 proved inefficient.

 In addition to the regulation of ITK activation by SH3/SH2 intermolecular interactions, ITK activation is also regulated by intermolecular interactions mediated by its PH domain. The PH domain of ITK is critical for TCR-induced recruitment to and association with the cell membrane [14]. Previous studies have suggested that cytoplasmic monomeric ITK becomes recruited to the cell membrane where it forms dimers and/or higher order multimers through intermolecular interactions regulated by the PH domain [26, 29]. Furthermore, data from Huang et al. have suggested that the PH domain-mediated aggregation of ITK might be regulated by membrane phospholipids [26]. The observation that the PH domain is critical for ITK membrane recruitment [14], whereas disruption of SH3-SH2 intermolecular interaction does not affect recruitment (Figure 4), suggests that the two types of interaction may regulate ITK activation through different mechanisms. How the intermolecular associations regulated by the PH domain influence the events leading to ITK activation is currently under investigation.

 Mutational analysis constitutes a powerful approach for establishing structure-function relationships in biological molecules. Very often a given mutation is designed to completely abolish a site of posttranslational modification. A case in point is the ITK Y511F mutant studied here. The biochemical effects of Y511 mutagenesis have been previously reported [15, 22], yet the effects of this mutation on Th2 cytokines that are known to be regulated by ITK [7] have not been specifically addressed before. As expected, total disruption of ITK phosphorylation on tyrosine 511 by mutation to phenylalanine affects phosphorylation of the downstream target, tyrosine 783 of PLC*γ*1, thus establishing this phenomenon in a physiologically relevant context. In view of the observed properties of this mutant, its effect on the Th2 signature cytokines IL-4 and IL-13 is not surprising. A novel observation is that ITK-Y511F is fully capable of localizing to the T cell-APC contact site and of associating with the adaptor protein SLP-76. Thus, trans-phosphorylation of ITK appears to be either a later event that follows recruitment to the contact site and interaction with SLP-76, and/or the phosphorylation status of Y511 in ITK simply has no effect on the interactions required to stabilize ITK at the contact site and bound to SLP-76.

 In contrast to the Y511F mutant, the ITK-BTK_(SH3) _ variant represents a significantly more complex mutational approach. Unlike posttranslational modifications that can be abrogated by a single amino acid change, protein-protein interactions involve numerous residues and often multiple protein interaction surfaces overlap on the surface of a single protein. The ITK-BTK_(SH3) _ variant was designed to abolish one protein-protein interaction (that between ITK SH3 and SH2) while maintaining the canonical ligand binding characteristic of the SH3 domain (binding to the proline-rich site on SLP-76). Even though *in vitro* experiments supported this approach [13], the present studies using nucleofection of ITK-BTK_(SH3) _ into primary lymphoid cells showed a different outcome; namely, the BTK SH3 domain substituted into the ITK protein does not maintain canonical ligand binding and thus results in diminished ITK signaling. Adding to the complexity associated with targeting protein interfaces by mutation, it is also important to note that intermolecular binding events are influenced by the cellular concentrations of the different protein species. Thus, variations in protein expression levels in different experimental systems have the potential to yield different outcomes.

 In conclusion, the data presented here and those previously published suggest that a number of different mechanisms may regulate the activation of ITK. In addition, as the details of intracellular signaling pathways are investigated by probing complex protein interaction interfaces, it will be important to consider how the experimental setup can influence the results.
